# Antibody-drug conjugates in breast cancer treatment: resistance mechanisms and the role of therapeutic sequencing

**DOI:** 10.20517/cdr.2024.180

**Published:** 2025-03-06

**Authors:** Émilie Audrey Larose, Xinying Hua, Silin Yu, Amritha Thulaseedharan Pillai, Zongbi Yi, Haijun Yu

**Affiliations:** ^1^Department of Radiation and Medical Oncology, Hubei Key Laboratory of Tumor Biological Behaviors, Hubei Cancer Clinical Study Center, Zhongnan Hospital of Wuhan University, Wuhan 430071, Hubei, China.; ^2^Wuhan Britain-China School, Wuhan 430071, Hubei, China.; ^3^School of Medicine, Wuhan University, Wuhan 430071, Hubei, China.; ^#^Authors contributed equally.

**Keywords:** Antibody-drug conjugates, breast cancer, resistance mechanisms, therapeutic sequencing, combination therapy, targeted treatment

## Abstract

Antibody-drug conjugates (ADCs) are a transformative approach in breast cancer therapy, offering targeted treatment with reduced toxicity by selectively delivering cytotoxic agents to cancer cells. While ADCs like trastuzumab emtansine (T-DM1), trastuzumab deruxtecan (T-DXd), and sacituzumab govitecan have shown significant efficacy, resistance mechanisms such as antigen loss, impaired internalization, and efflux of cytotoxic payloads challenge their effectiveness. This review discusses these resistance mechanisms and explores advanced strategies to overcome them, including innovations in linker chemistry, multi-antigen targeting, and biomarker-driven personalization. Additionally, therapeutic sequencing - determining the optimal order of ADCs with other treatments such as chemotherapy, endocrine therapy, and immunotherapy - is examined as a crucial approach to maximize ADC efficacy and manage resistance. Evidence-based sequencing strategies, particularly for human epidermal growth factor receptor 2 (HER2)-positive and triple-negative breast cancer (TNBC), are supported by clinical trials demonstrating the benefits of ADCs in both early-stage and metastatic settings. The potential of combination therapies, such as ADCs with immune checkpoint inhibitors (ICIs), further highlights the evolving landscape of breast cancer treatment. As ADC technology advances, personalized approaches integrating biomarkers and optimized sequencing protocols offer promising avenues to enhance treatment outcomes and combat resistance in breast cancer.

## INTRODUCTION

Antibody-drug conjugates (ADCs) have significantly impacted breast cancer treatment by merging targeted monoclonal antibody therapy with cytotoxic drugs to kill cancer cells while selectively minimizing harm to healthy tissue^[1]^. This targeted approach aligns with Paul Ehrlich’s early concept of a “magic bullet” - a therapeutic that could precisely target and eliminate disease-causing cells^[2,3]^. By combining monoclonal antibodies that recognize specific antigens on cancer cells with potent cytotoxic payloads, ADCs are designed to deliver high concentrations of chemotherapy directly to tumor cells, thus minimizing systemic toxicity and improving therapeutic outcomes^[4,5]^. ADCs are particularly transformative for breast cancer, where different subtypes - human epidermal growth factor receptor 2 (HER2) -positive, triple-negative, and hormone receptor-positive (HR+) - each require distinct treatment strategies^[6]^. ADCs offer a promising solution for targeting specific tumor profiles within these subtypes, bringing a new level of precision to breast cancer therapy^[7,8]^.

The landscape of breast cancer treatment has evolved to incorporate ADCs across multiple subtypes, each with unique molecular profiles that influence treatment responses. HER2-positive breast cancer, characterized by overexpression of the HER2 receptor, has been a primary focus of ADC therapy due to the availability of highly specific targets for antibody binding. The regimen combining pyrotinib, trastuzumab, and docetaxel has shown significant promise as a first-line treatment option for patients with HER2-positive breast cancer^[9]^. Trastuzumab emtansine (T-DM1), the first FDA-approved ADC for HER2-positive metastatic breast cancer, marked a significant advancement by showing efficacy in patients previously treated with trastuzumab and other chemotherapies^[10]^. Clinical trials, such as the EMILIA trial, have demonstrated T-DM1’s efficacy and safety, setting a foundation for ADCs as a mainstay in HER2-positive breast cancer treatment^[11,12]^. Building on this success, trastuzumab deruxtecan (T-DXd), with a higher drug-to-antibody ratio and a cleavable linker for efficient drug release within the tumor, has provided enhanced effectiveness in HER2-positive cases by minimizing off-target effects^[13,14]^.

A significant advancement in ADC treatment is the expansion of its applicability to breast cancer patients with HER2-low expression, broadening therapeutic options beyond the traditional HER2-positive subset^[6,15]^. Triple-negative breast cancer (TNBC), known for its aggressive behavior and limited targeted treatment options, has also benefited from ADCs. TNBC lacks estrogen receptor (ER), progesterone receptor (PR), and HER2 expression, making traditional endocrine and HER2-targeted therapies ineffective. Sacituzumab govitecan, an ADC targeting trophoblast cell surface antigen 2 (TROP-2), represents a novel treatment for TNBC, significantly improving survival outcomes for patients with advanced disease^[16,17]^. This ADC approach in TNBC showcases ADCs’ adaptability in addressing various breast cancer subtypes, offering new therapeutic options for challenging cases^[4,18]^.

HR+ breast cancer, the most common breast cancer subtype, also stands to benefit from ongoing ADC research^[19]^. Although HR+ tumors traditionally respond to endocrine therapy, some patients experience recurrence and progression despite treatment. ADCs targeting antigens specific to HR+ tumors are currently under investigation, expanding ADCs’ potential applications across all major breast cancer subtypes^[19,20]^.

Despite the progress ADCs have made in breast cancer treatment, resistance remains a significant challenge. Understanding these mechanisms is essential to develop strategies that prolong ADCs’ efficacy. Resistance can arise from intrinsic factors within the tumor microenvironment (TME) or acquired adaptations in response to ADC exposure. One prominent mechanism involves downregulation or loss of the target antigen, which reduces the ADC’s ability to bind effectively to cancer cells, thereby limiting its therapeutic impact^[21,22]^. This issue is compounded by tumor heterogeneity, where variations in antigen expression across tumor cells lead to inconsistent targeting, undermining the efficacy of ADCs that rely on uniform antigen presence^[23,24]^.

Other resistance mechanisms are linked to disruptions in ADC internalization and intracellular processing. Once bound to the target antigen, ADCs are internalized by the cancer cell, where they undergo lysosomal degradation to release the cytotoxic payload. However, impairments in this process - such as reduced endocytosis or lysosomal function - can prevent the payload from reaching its intended intracellular target, diminishing ADC effectiveness^[3,25,26]^. Additionally, resistance to the cytotoxic payload itself poses challenges. Cancer cells may upregulate drug efflux transporters, such as multidrug resistance protein 1 (MDR1), which actively remove the cytotoxic agent from the cell, lowering its intracellular concentration and reducing its cytotoxic effects^[22,27]^.

Further research has revealed mutations in intracellular targets and alterations in apoptotic pathways, which can also decrease ADC efficacy. These adaptations allow cancer cells to withstand the cytotoxic payload’s effects, complicating ADC therapy’s long-term success^[28,29]^. The complexity of these resistance mechanisms underscores the need for ongoing research and innovation in ADC design to improve treatment durability and patient outcomes.

To address the challenges posed by resistance, researchers are exploring advancements in ADCs’ design, including the development of bispecific and dual-target ADCs, as well as improvements in linker technology. Bispecific ADCs, which can target two different antigens, offer a promising approach to overcoming antigen heterogeneity by ensuring that even if one target antigen is downregulated, the ADC can still bind to the alternate antigen^[14,23]^. In addition, innovative linker chemistries, such as cleavable linkers that release the payload in specific intracellular conditions, enhance drug delivery efficiency and address some resistance issues related to drug release^[4,30]^.

Therapeutic sequencing - the order and timing in which ADCs are administered relative to other treatments - is another strategy that may help extend ADC efficacy. Early studies suggest that introducing ADCs earlier in treatment pathways, particularly in HER2-positive and TNBC cases, may prevent resistance from developing as rapidly as it might in later-line treatments. ADCs may also work synergistically with other therapies, such as immune checkpoint inhibitors (ICIs), to enhance antitumor immune responses and reduce resistance development^[8]^. Personalizing ADC therapy using biomarkers to identify patients likely to respond to specific ADCs is essential in optimizing therapeutic outcomes^[28]^.

This review aims to analyze the mechanisms of resistance that limit the effectiveness of ADCs in breast cancer and to explore therapeutic sequencing strategies that could maximize ADC efficacy and patient survival. By examining the roles of ADCs across different breast cancer subtypes, understanding how resistance mechanisms emerge, and evaluating sequencing strategies, this review seeks to provide a comprehensive overview of ADC therapy in breast cancer. As the field of ADCs advances, ongoing research focused on overcoming resistance through innovative designs, combination strategies, and personalized treatment will be essential to fully realize ADCs’ potential in breast cancer management.

## CURRENT ADC LANDSCAPE IN BREAST CANCER

Developing ADCs has revolutionized breast cancer therapy by combining selective targeting with potent cytotoxic agents. ADCs deliver chemotherapy directly to cancer cells while minimizing harm to normal tissues, a significant advantage over traditional chemotherapy. This section delves into the structure and mechanisms of ADCs, details on approved ADCs in breast cancer, and their application in early and metastatic breast cancer settings.

### ADC structure and mechanism of action

ADCs are comprised of three primary components: a monoclonal antibody (mAb) that binds to specific antigens on cancer cells, a linker that connects the antibody to a cytotoxic payload, and the payload itself. The monoclonal antibody enables selective targeting, which is crucial for minimizing off-target effects. The linker, which can be either cleavable or non-cleavable, ensures that the cytotoxic payload remains intact until the ADC reaches the targeted cancer cell. This stability prevents premature drug release, thereby reducing systemic toxicity. Upon reaching the cancer cell, the linker is cleaved to release the cytotoxic agent directly within the cell, maximizing the therapeutic impact of the payload^[22,24,31,32]^.

**Figure 1:**
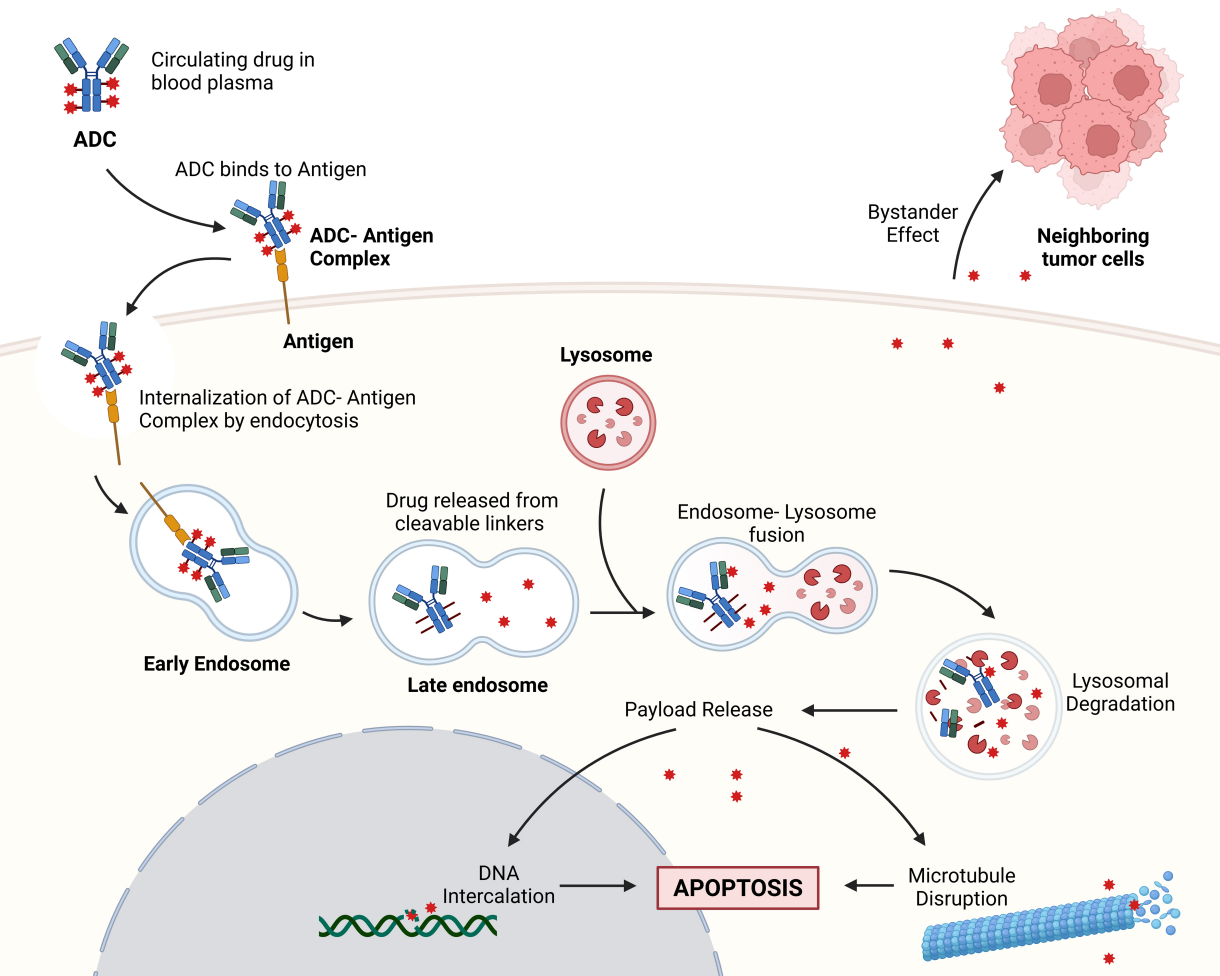
Mechanism of action of ADCs. ADCs bind to target antigens on cancer cells, forming an ADC-antigen complex, which is internalized via endocytosis^[5]^. Within endosomes, cleavable linkers release the cytotoxic payload upon fusion with lysosomes, triggering mechanisms like DNA intercalation and microtubule disruption that lead to apoptosis. Additionally, the bystander effect allows the cytotoxic payload to impact neighboring tumor cells, enhancing therapeutic efficacy. Created in BioRender. Larose, E. (2024) https://BioRender.com/f12z842. ADCs: Antibody-drug conjugates.

The mechanism of action of ADCs begins with the binding of the mAb to specific antigens expressed on the surface of cancer cells. This binding facilitates the internalization of the ADC through endocytosis, a process that is critical for subsequent drug release. Once inside the cell, the linker is cleaved - often by lysosomal enzymes such as cathepsin B - releasing the cytotoxic payload directly into the intracellular environment^[33,34]^. The released drug can then disrupt vital cellular processes, such as microtubule dynamics or DNA replication, ultimately leading to apoptosis of the targeted cancer cell^[35,36]^. This mechanism is particularly advantageous over conventional chemotherapy, which lacks the specificity afforded by the targeting capabilities of ADCs^[31]^. The stability of the linker in ADCs is crucial for ensuring the payload is only released upon reaching the target cell, with cleavable linkers responding to specific intracellular conditions, such as pH changes or enzyme presence, and non-cleavable linkers requiring complete ADC degradation for drug release^[5,33]^. Additionally, the bystander effect enhances ADC efficacy by allowing the cytotoxic payload to impact neighboring tumor cells, which is particularly beneficial in heterogeneous tumors^[29,36]^. Figure 1 provides a visual representation of the ADC mechanism of action, highlighting the pathway from ADC binding to the cancer cell surface through to internalization and intracellular cytotoxic payload release.

### Approved ADCs in breast cancer

Several ADCs have been approved for breast cancer, including T-DM1, T-DXd, and sacituzumab govitecan, each targeting specific subtypes. T-DM1 combines trastuzumab, a monoclonal antibody against HER2, with the cytotoxic agent emtansine, a tubulin inhibitor. T-DM1 was approved following the results of the EMILIA trial, which demonstrated improved progression-free survival (PFS) and overall survival (OS) in HER2-positive metastatic breast cancer patients previously treated with trastuzumab and chemotherapy^[10,11,29,37]^. The targeted delivery of emtansine in T-DM1 minimizes systemic toxicity, allowing for higher doses directly to cancer cells, thus enhancing its therapeutic index and establishing its efficacy in HER2-positive tumors with reduced side effects compared to traditional chemotherapy^[38-40]^.

T-DXd is a next-generation ADC with a high drug-to-antibody ratio and a cleavable linker, facilitating efficient drug release within the TME and allowing for a bystander effect that targets adjacent cancer cells - an advantage in heterogeneous tumors where antigen expression may vary^[41-43]^. T-DXd has demonstrated significant efficacy in HER2-positive metastatic breast cancer patients who have progressed on prior HER2 therapies and has shown promising activity in HER2-low breast cancers, thus expanding its therapeutic potential beyond traditional HER2-positive cases^[13,29]^.

Sacituzumab govitecan, targeting TROP-2, has shown significant efficacy in TNBC, a challenging subtype lacking HER2, ER, and PR expression, with studies demonstrating survival benefits and a bystander effect that enhances its therapeutic reach^[17,44-47]^.

ADCs provide significant advantages over traditional chemotherapy due to their targeted delivery, which selectively binds to cancer cell antigens and reduces exposure to normal tissues, thereby minimizing systemic toxicity and side effects^[4,5]^. Leveraging the “bystander effect” to impact adjacent cancer cells, ADCs like T-DM1, T-DXd, and sacituzumab govitecan offer a precision approach that allows higher, more effective doses of cytotoxic drugs while highlighting their clinical potential to transform breast cancer treatment. Table 1 summarizes the key characteristics of FDA-approved ADCs for breast cancer treatment, including the target antigen, type of cytotoxic payload, linker technology, approved clinical indications, pivotal clinical trials, and year of approval.

**Table 1:** Summary of FDA-approved ADCs for breast cancer

**ADC name**	**Target antigen**	**Payload type**	**Linker type**	**Approved indications**	**Key clinical trial(s)**	**Year of approval**
T-DM1	HER2	DM1 (microtubule inhibitor)	Non-cleavable linker	HER2-positive metastatic breast cancer after prior therapy with trastuzumab and a taxane	EMILIA^[11]^	2013
T-DXd	HER2	Topoisomerase I inhibitor	Cleavable linker (tetrapeptide-based)	HER2-positive and HER2-low metastatic breast cancer following multiple prior lines of HER2-targeted therapy	DESTINY-Breast01, DESTINY-Breast03^[48]^	2019
Sacituzumab govitecan	TROP-2	SN-38 (topoisomerase I inhibitor)	Cleavable linker	TNBC after at least two prior therapies in the metastatic setting	ASCENT^[44]^	2020
Dato-DXd	TROP-2	Topoisomerase I inhibitor	Cleavable linker	TNBC and HR+/HER2- metastatic breast cancer	TROPION-Breast01^[49]^	2024
SKB264 (MK-2870)	TROP-2	Topoisomerase I inhibitor	Cleavable linker	Advanced TNBC and HR+/HER2- metastatic breast cancer	NCT03729596^[50]^	2024

FDA: ADCs: antibody-drug conjugates; T-DM1: trastuzumab emtansine; HER2: human epidermal growth factor receptor 2; T-DXd: trastuzumab deruxtecan; TROP-2: trophoblast cell surface antigen 2; TNBC: triple-negative breast cancer; Dato-DXd: datopotamab deruxtecan; HR+: hormone receptor-positive; SKB264 (MK-2870): sacituzumab tirumotecan.

### ADCs in early *vs.* metastatic settings

The application of ADCs in breast cancer treatment has evolved significantly, moving from a focus on metastatic settings to an expanding role in early-stage disease management. This transition is underscored by pivotal trials such as the KATHERINE trial, which demonstrated that adjuvant T-DM1 significantly improved invasive disease-free survival (iDFS) for HER2-positive early breast cancer patients with residual disease following neoadjuvant therapy, providing a more effective option with fewer side effects than standard chemotherapy^[4,23,51]^. Additionally, ongoing research on T-DXd and sacituzumab govitecan in high-risk early-stage patients aims to reduce disease progression and improve survival outcomes, reflecting a shift toward targeted therapies with minimized systemic toxicity^[3,4,20]^.

In metastatic breast cancer, ADCs such as T-DM1, T-DXd, and sacituzumab govitecan, have demonstrated substantial efficacy, improving PFS and OS in heavily pretreated patients. For instance, sacituzumab govitecan has shown promising results in TNBC, an aggressive subtype with limited treatment options, yielding improved PFS and OS compared to traditional chemotherapy^[48]^. However, resistance mechanisms, including decreased antigen expression, impaired internalization, and increased efflux of the cytotoxic payload, present challenges to effective ADC use in metastaticy^[3,21]^. To address these issues, next-generation ADCs with optimized linkers and payloads are being developed, alongside trials investigating ADCs in combination with ICIs to enhance therapeutic responses and delay resistance^[8,49]^.

The integration of ADCs across both early and metastatic settings underscores their transformative potential in breast cancer management. The success of trials such as KATHERINE, along with ongoing studies on T-DXd and sacituzumab govitecan, highlights the effectiveness of ADCs in improving treatment outcomes with reduced side effects, particularly for patients at high risk of recurrence. As ADC research progresses, the development of next-generation ADCs and combination therapies may further enhance the therapeutic landscape, providing more effective and personalized treatment options for breast cancer patients^[15]^.

### Recent advances in ADCs for breast cancer: sacituzumab tirumotecan and datopotamab deruxtecan

Recent developments in ADCs have led to the emergence of sacituzumab tirumotecan [SKB264 (MK-2870)] and datopotamab deruxtecan (Dato-DXd), which show promise in treating TNBC and HER2-low-expressing breast cancer. These novel ADCs leverage improved linker technology and potent cytotoxic payloads to enhance treatment efficacy while minimizing off-target effects.

### SKB264 (MK-2870)

SKB264 is a TROP2-directed ADC that utilizes a next-generation topoisomerase I inhibitor payload, designed to improve stability and tumor selectivity. This ADC binds to TROP2-expressing tumor cells, facilitating internalization and intracellular release of the cytotoxic payload^[52]^. The Phase II trial (NCT04152499) of SKB264 in TNBC reported an objective response rate (ORR) of 43.8% and a median PFS of 7.4 months, highlighting its potential as a second-line therapy^[53]^. Compared to sacituzumab govitecan, SKB264 exhibits lower toxicity and improved durability, making it a promising candidate for future therapeutic sequencing.

### Dato-DXd

Dato-DXd is a TROP2-targeted ADC with a proprietary deruxtecan payload, designed to enhance DNA damage responses in tumor cells. Like T-DXd, Dato-DXd leverages a cleavable linker, allowing bystander effect-mediated cytotoxicity^[54]^. The Phase I TROPION-PanTumor01 study (NCT03401385) demonstrated a 35% ORR in previously treated HR+/HER2-low breast cancer patients, with durable responses lasting over 7 months^[55]^. Given its encouraging monotherapy activity, Dato-DXd is being evaluated in combination with ICIs and CDK4/6 inhibitors for HR+ breast cancer.

### Implications for breast cancer treatment

Both SKB264 and Dato-DXd represent the next generation of ADCs, expanding treatment options for HER2-low and TNBC patients. Their improved payloads, linker technology, and favorable safety profiles position them as strong candidates for combination therapies and novel treatment sequencing strategies. Ongoing trials will further define their optimal use in clinical practice, particularly in patients with ADC resistance or prior chemotherapy exposure.

The continuous advancement of ADC technology has led to the approval of new ADCs targeting breast cancer. Song *et al.* (2024) discussed the impact of crotonylation of minichromosome maintenance complex component 6 (MCM6) in enhancing chemotherapy sensitivity via DNA replication stress, which has implications for improving ADC efficacy^[56]^. Additionally, Shao *et al.* (2024) evaluated the pharmacokinetics and efficacy of Inetetamab in combination with vinorelbine for HER2-positive metastatic breast cancer, highlighting its comparable effectiveness in weekly and three-weekly regimens^[57]^.

## MECHANISMS OF RESISTANCE TO ADCS [FIGURE 2]

**Figure 2:**
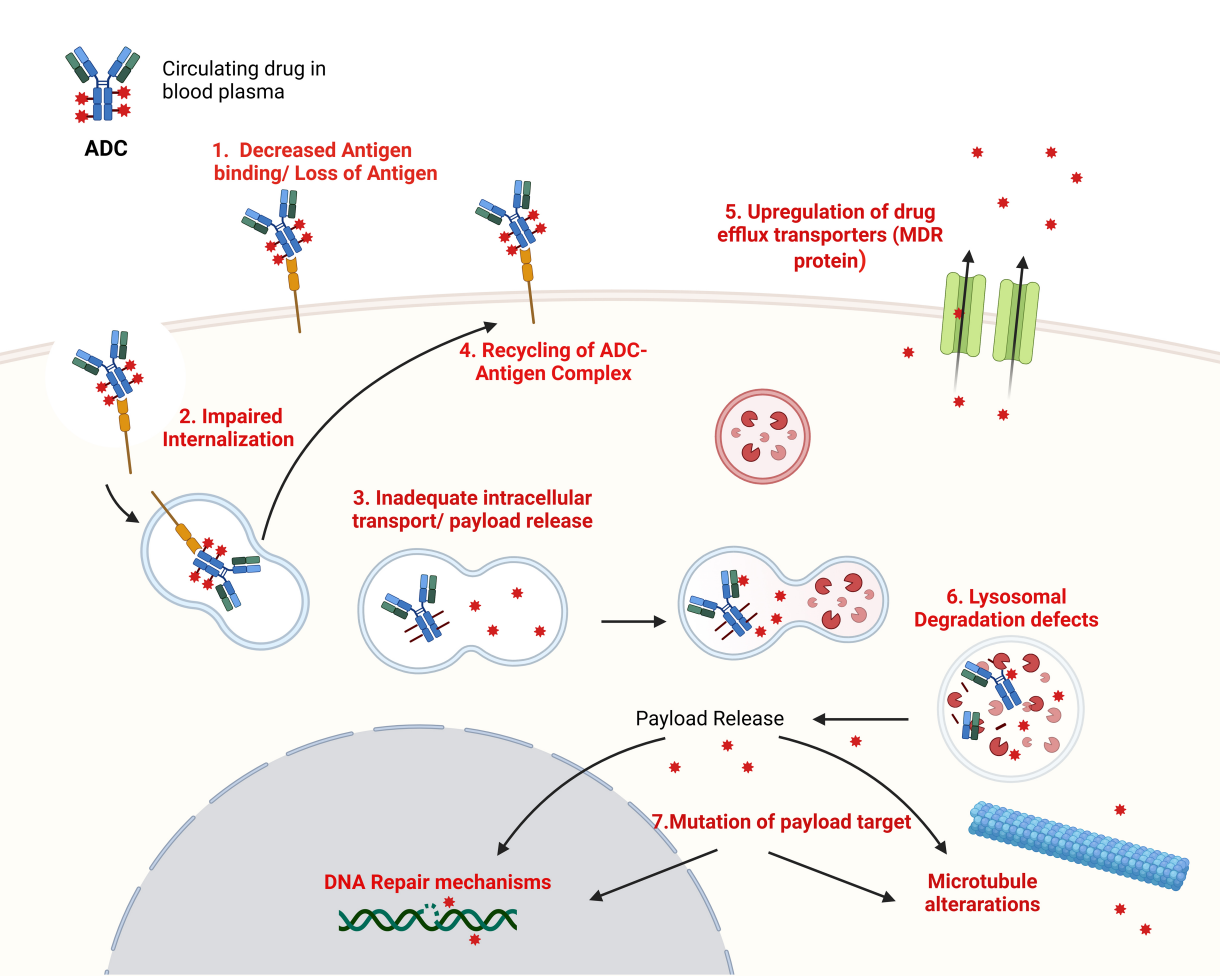
Mechanisms of resistance to ADCs. Resistance mechanisms include antigen loss or (1) decreased antigen binding, (2) impaired internalization, (3) inadequate intracellular trafficking leading to reduced payload release and (4) recycling of the ADC-antigen complex. Additional resistance pathways involve (5) upregulation of drug efflux transporters (e.g., MDR, Pgp), (6) lysosomal degradation defects, and (7) mutations in the payload target, all of which diminish ADC therapeutic efficacy. Created in BioRender. Larose, E. (2024) https://BioRender.com/f12z842. ADCs: Antibody-drug conjugates; MDR: multidrug resistance; Pgp: P-glycoprotein.

ADCs offer a targeted therapeutic approach in cancer treatment, particularly in breast cancer, by delivering cytotoxic drugs directly to tumor cells while minimizing exposure to healthy cells. Despite their potential, resistance mechanisms limit ADC efficacy, and understanding these barriers is crucial for advancing ADC technology. Recent studies have identified multiple resistance mechanisms to ADCs in breast cancer. Saleh *et al.* (2024)^[58]^ provided insights into how HER2-positive and HER2-low breast cancers develop distinct resistance patterns, emphasizing the role of antigen shedding, altered intracellular trafficking, and efflux pumps. Zou *et al.* (2024) identified crVDAC3 as a critical factor in T-DXd resistance by impeding HSPB1 ubiquitination, suggesting that ferroptosis plays a significant role in ADC resistance in HER2-low breast cancer^[59]^.

### Antigen loss and heterogeneity

Antigen loss and tumor heterogeneity are significant challenges in ADC therapy for breast cancer, as they impact the targeted delivery of cytotoxic agents and contribute to resistance. A primary mechanism of resistance involves the downregulation or complete loss of target antigens, reducing the effectiveness of ADCs by limiting selective binding. This adaptive mechanism can arise through genetic mutations, epigenetic alterations, or selective expansion of antigen-negative cells, allowing tumors to evade ADC effects^[3,25,60]^. This phenomenon is particularly evident in HER2-positive breast cancer, where ADCs like T-DM1 and T-DXd rely on HER2 expression. Studies have shown that resistant cells often exhibit reduced HER2 levels, diminishing ADC efficacy and underscoring the need for continuous monitoring of antigen expression during treatment to adapt strategies^[4,29,50]^.

Tumor heterogeneity further complicates ADC effectiveness. Variability in antigen expression within tumors or between metastatic sites can result in inconsistent ADC binding, allowing cells with low or absent target antigens to survive, potentially leading to relapse^[3,60]^. For instance, HER2-positive tumors often contain cells with varying HER2 expression levels, which can reduce the efficacy of HER2-targeting ADCs^[2,8]^. Addressing heterogeneity may require the development of multi-targeting ADCs or combination therapies that integrate ADCs with other modalities, broadening targeting capabilities across diverse tumor cell populations^[61,62]^.

To counteract these resistance mechanisms, researchers are exploring strategies like dual payload ADCs, which deliver multiple cytotoxic agents targeting different pathways within tumor cells, potentially enhancing therapeutic effects and reducing resistance likelihood^[5,62]^. Additionally, combining ADCs with ICIs may amplify the immune response against heterogeneous tumor populations, helping to address antigen loss^[3,60]^. Advances in site-specific conjugation techniques are also being investigated to improve drug-to-antibody ratios, consistency, and pharmacokinetics, potentially enhancing therapeutic indices^[63,64]^.

### Impaired ADC internalization and trafficking

Impaired internalization and trafficking are major challenges to the efficacy of ADCs in breast cancer treatment, as these therapies depend on the successful internalization of the ADC-antigen complex and trafficking to lysosomes for payload release. Alterations in endocytic and lysosomal pathways can prevent effective ADC function by limiting internalization or hindering payload release, which leads to resistance. Cancer cells may adapt by reducing endocytic receptor expression or modifying endocytic pathways, preventing effective ADC internalization and diminishing therapeutic efficacy, a mechanism observed in breast cancer resistance models^[25,29,65]^.

Once internalized, ADCs rely on lysosomal trafficking for payload release, a process that can be compromised by deficiencies in lysosomal function. Effective linker cleavage and drug release are contingent on lysosomal proteolytic activity; without it, the cytotoxic payload may remain bound to the antibody, allowing cancer cells to survive despite ADC attachment^[4,61]^. Linker stability between the antibody and payload also plays a pivotal role in ADC function, as linkers must remain stable in circulation yet cleave efficiently within lysosomes to enable intracellular drug release. In resistant cancer cells, linker cleavage can be hindered by altered lysosomal conditions or excessive linker stability, reducing ADC potency^[3]^.

To address these limitations, novel linker designs such as pH-sensitive or enzyme-responsive linkers are being developed to enhance payload release under specific intracellular conditions, potentially overcoming resistance linked to impaired linker cleavage^[64,66]^. Advancements in understanding endocytic and lysosomal pathways, alongside innovative linker designs, aim to improve ADC efficacy, offering more effective treatments for patients with breast cancer^[67-70]^.

### Payload-related resistance

Resistance to ADCs in breast cancer can stem from cellular adaptations that reduce the effectiveness of the cytotoxic payload. A prominent mechanism involves the upregulation of drug efflux transporters, such as MDR1, which actively pumps cytotoxic agents out of cells, reducing intracellular concentrations and weakening therapeutic impact^[4,29]^. Increased MDR1 expression has been observed in ADC-resistant breast cancer cells, particularly those subjected to long-term treatment, allowing these cells to expel the payload more rapidly than it can act. Strategies to counter this resistance include using efflux transporter inhibitors alongside ADCs or designing payloads less prone to efflux mechanisms, though these approaches require careful balancing to avoid unintended toxicity from enhanced payload retention.

Another form of payload-related resistance involves mutations or modifications within the intracellular targets of ADC payloads. Many ADCs utilize tubulin inhibitors to disrupt cell division by targeting microtubules, yet mutations in tubulin or associated proteins can prevent effective binding and render these agents ineffective^[6]^. Similarly, ADCs that employ topoisomerase inhibitors rely on intact topoisomerase enzymes to induce DNA damage and initiate cell death, but mutations or post-translational modifications in topoisomerase can reduce the enzyme’s affinity for the payload, diminishing its cytotoxic potential^[6]^. To overcome such resistance, researchers are exploring alternative cytotoxic agents and multidrug ADCs that target multiple pathways, ensuring efficacy even if one pathway is compromised^[3,71]^.

Novel strategies to counter payload-related resistance focus on designing payloads less susceptible to efflux mechanisms and engineering linkers that release the payload more effectively under specific intracellular conditions. For instance, pH-sensitive or enzyme-responsive linkers are being developed to release the payload in the acidic environments of tumors or in response to particular enzymatic activities within cancer cells, thus enhancing ADC efficacy in resistant cells^[71,72]^. These advancements, alongside innovative approaches in payload design and combination therapies, represent a promising direction in addressing payload-related resistance in ADCs for breast cancer, aiming to improve treatment outcomes^[25]^.

### TME and ADC resistance

The TME plays a pivotal role in shaping the efficacy and resistance of ADCs. Several key components within the TME, including cancer-associated fibroblasts (CAFs), tumor-associated macrophages (TAMs), CD8+ T cells, and natural killer (NK) cells, influence ADC function by modulating drug penetration, altering antigen expression, and regulating immune responses^[73]^.

#### CAFs and ADC penetration

CAFs are key stromal components that contribute to ADC resistance by producing extracellular matrix (ECM) proteins, which impede ADC penetration into tumors^[74]^. The dense collagen network formed by CAFs can physically block ADC diffusion, reducing drug delivery efficiency^[75]^. CAF-secreted TGF-β also downregulates HER2 expression in breast cancer cells, decreasing ADC binding and internalization^[71]^. Strategies such as CAF-targeted therapies (e.g., TGF-β inhibitors) and ECM-modifying agents are being investigated to improve ADC penetration and efficacy.

#### TAMs and ADC clearance

TAMs contribute to ADC resistance by enhancing antigen shedding and ADC degradation within the TME^[76]^. TAMs express Fc gamma receptors (FcγRs), which promote ADC uptake and lysosomal degradation, reducing ADC availability for tumor targeting^[77]^. Additionally, TAMs secrete immunosuppressive cytokines (e.g., IL-10, TGF-β) that dampen CD8+ T cell responses, limiting the immune-enhancing effects of ADC-induced tumor cell death^[78]^. Macrophage-targeted therapies (e.g., CSF1R inhibitors) are being explored to deplete TAMs and restore ADC efficacy.

#### CD8+ T cells and ADC-induced immunogenic cell death

ADCs can enhance antitumor immunity through immunogenic cell death (ICD), leading to the release of tumor-associated antigens (TAAs) and damage-associated molecular patterns (DAMPs)^[79]^. Effective ADCs stimulate CD8+ T cell priming, enhancing tumor antigen presentation via dendritic cells (DCs)^[3,25,79-81]^. However, TME-mediated T cell exhaustion, driven by chronic antigen exposure and checkpoint inhibition (e.g., PD-1/PD-L1 upregulation), can diminish the immune-enhancing effects of ADCs^[67]^. Combining ADCs with ICIs (e.g., anti-PD-1, anti-CTLA-4) has been proposed as a strategy to revitalize CD8+ T cell responses.

#### NK cells and antibody-dependent cellular cytotoxicity

ADCs with Fc-mediated activity can engage NK cells via FcγRIII (CD16), leading to antibody-dependent cellular cytotoxicity (ADCC)^[68-70]^. The effectiveness of NK cell-mediated ADCC is often compromised in immune-suppressive TMEs, where high levels of TGF-β and PD-L1 expression inhibit NK cell function^[82]^. Enhancing NK cell activation via IL-15-based therapies or combining ADCs with NK cell engagers may improve treatment responses in ADC-resistant tumors.

### T-DXd resistance mechanisms in HER2-low expression *vs.* HER2-positive breast cancer

T-DXd has demonstrated superior efficacy in both HER2-positive and HER2-low-expressing breast cancer compared to earlier ADCs like T-DM1. However, resistance mechanisms differ between these two breast cancer subtypes due to variations in HER2 expression levels, tumor heterogeneity, and distinct tumor microenvironmental influences^[58,82]^.

#### HER2 expression levels and antigen shedding

In HER2-positive breast cancer, high HER2 expression provides more binding sites for T-DXd, leading to efficient ADC internalization and payload release^[56]^. Resistance in HER2-positive cases often arises from HER2 downregulation or gene mutations, which reduce ADC binding and internalization^[57]^. In HER2-low expression breast cancer, HER2 expression is already low, making further downregulation a more pronounced resistance mechanism. Some tumors shed HER2 extracellularly, further limiting ADC targeting^[83]^.

#### Extracellular protease activity and payload release

A unique resistance mechanism in HER2-low expression tumors involves TME-derived proteases, such as cathepsin L. In HER2-low expression tumors, cathepsin L can prematurely cleave T-DXd’s linker, leading to payload release before ADC internalization, reducing its intracellular cytotoxic efficacy^[83]^. In contrast, HER2-positive tumors internalize T-DXd more efficiently, making intracellular resistance mechanisms (e.g., lysosomal dysfunction) more prominent^[58]^.

#### The bystander killing effect and tumor heterogeneity

T-DXd’s cytotoxic payload exhibits a bystander killing effect, where neighboring HER2-negative cells can be affected even if they do not directly bind T-DXd^[59]^. In HER2-positive tumors, this effect is less crucial because ADC penetration and uptake are already high. However, in HER2-low-expressing tumors, bystander killing is a major mechanism compensating for the limited direct binding^[83]^. Tumors that increase drug efflux transporter expression (e.g., Breast cancer resistance protein ABCG2/BCRP) or develop enhanced DNA damage repair mechanisms become resistant by neutralizing the cytotoxic effect of T-DXd’s payload before it can spread via the bystander effect^[58]^.

### Strategies to overcome resistance

Given the distinct resistance mechanisms in HER2-low expression versus HER2-positive breast cancer, tailored strategies are being explored. For HER2-positive resistance, strategies targeting HER2 mutations and HER2 shedding prevention (e.g., HER2 re-sensitizing agents) may help maintain ADC efficacy. Additionally, lysosomal function modulators are being investigated to counteract impaired payload release. In cases of HER2-low-expressing resistance, protease inhibitors (e.g., cathepsin L inhibitors) are being explored to prevent premature T-DXd cleavage. Enhancing bystander killing efficiency through synergistic combinations with DNA damage response inhibitors might also improve ADC efficacy.

## STRATEGIES TO OVERCOME RESISTANCE [FIGURE 3]

**Figure 3:**
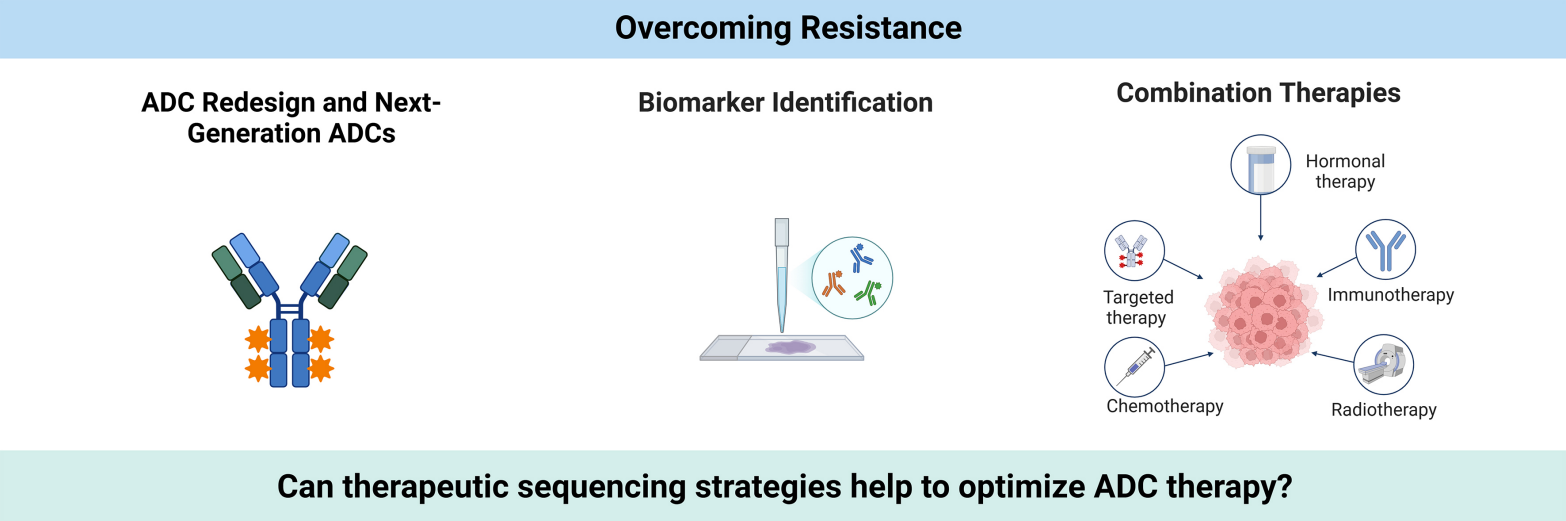
Strategies for overcoming resistance in ADC therapy. The illustration highlights three key approaches: redesigning ADCs with next-generation linkers and payloads, identifying biomarkers for personalized treatment, and implementing combination therapies with endocrine therapy, immunotherapy, chemotherapy, and radiotherapy. These strategies aim to optimize therapeutic outcomes and address resistance mechanisms. Created in BioRender. Larose, E. (2024) https://BioRender.com/f12z842. ADC: Antibody-drug conjugate.

Overcoming resistance to ADCs is essential to maximize their therapeutic potential in breast cancer treatment. Advances in ADC design, personalized approaches using biomarkers, and combination therapies are three primary strategies to enhance ADC effectiveness and address resistance mechanisms. This section explores innovations in ADC development, biomarker-driven personalization, and the potential of combined treatment approaches to counteract resistance.

### ADC redesign and next-generation ADCs

One of the most promising strategies to enhance ADC efficacy is through innovations in linker chemistry, a critical component that connects the antibody to the cytotoxic payload. Linkers must remain stable in circulation to prevent premature drug release yet cleave effectively within the target cell to release the payload. Next-generation ADCs are focused on cleavable linkers that respond to specific intracellular conditions, such as acidic pH or enzyme activity, which can enhance ADC efficacy and reduce off-target effects^[3,4,27]^. For instance, targeted release mechanisms help prevent premature drug delivery, thereby promoting optimal cytotoxicity specifically within tumor cells^[14,84]^.

Targeting multiple antigens is another promising approach to combat resistance, particularly due to antigen loss or tumor heterogeneity. Bispecific ADCs can bind to two distinct antigens, ensuring effective targeting even if one antigen is downregulated. This dual-targeting strategy is especially beneficial in heterogeneous tumors, enhancing the range of tumor cells targeted and improving therapeutic efficacy^[3,4]^. The development of dual-target ADCs represents a critical advancement, as it enables a broader scope of tumor eradication by reaching cells with varying antigen expression levels^[84]^.

### Biomarker identification and personalized ADC therapy

The identification of predictive biomarkers is crucial for optimizing patient selection and improving the efficacy of ADCs in breast cancer treatment. While HER2 expression remains a key criterion for ADC eligibility, emerging biomarkers such as gene expression signatures, circulating tumor DNA (ctDNA), and tumor immune microenvironment factors are refining treatment strategies and guiding clinical decisions. Table 2 presents key biomarkers under investigation for predicting ADC efficacy and resistance, which are critical for guiding personalized treatment strategies and enhancing the clinical success of next-generation ADCs.

**Table 2:** Biomarkers for predicting efficacy and resistance in ADC therapy for breast cancer

**Biomarker**	**Role in ADC response**	**Detection methods**	**Clinical trials/studies**	**Associated response**
HER2 expression	Predicts response to HER2-targeting ADCs (e.g., T-DM1, T-DXd); higher expression correlates with increased efficacy	IHC, FISH	EMILIA, DESTINY-Breast01^[48]^	Good
TROP-2 expression	High expression correlates with response to TROP-2–targeting ADCs like sacituzumab govitecan in TNBC	IHC, RNA-seq	ASCENT^[48]^	Good
MDR1 expression	Associated with resistance due to efflux of ADC payload, reducing intracellular drug levels	qPCR, Western blot, IHC	Preclinical studies on efflux resistance^[29]^	Poor
Tumor heterogeneity	Predicts variable ADC efficacy due to mixed antigen expression within tumors	NGS, single-cell RNA-seq	Studies on HER2 heterogeneity and response^[3]^	Poor

The table lists biomarkers that are being studied to predict patient response and potential resistance to ADCs, detailing their role in ADC efficacy, detection methods, and relevant clinical trials. These biomarkers support the personalization of ADC therapy and help in identifying patients most likely to benefit from treatment. ADC: Antibody-drug conjugate; HER2: human epidermal growth factor receptor 2; T-DM1: trastuzumab emtansine; T-DXd: trastuzumab deruxtecan; IHC: immunohistochemistry; FISH: TROP-2: trophoblast cell surface antigen 2; TNBC: triple-negative breast cancer; MDR1: multidrug resistance protein 1; NGS: next-generation sequencing.

#### HER2 expression and beyond

While HER2 levels guide the use of trastuzumab-based ADCs, heterogeneous HER2 expression across tumor cells can affect ADC efficacy. Quantitative HER2 scoring methods, including digital pathology and artificial intelligence (AI)-based HER2 assessment, are being developed to refine patient selection^[85]^. The HER2-low paradigm has expanded ADC eligibility, as ADCs like T-DXd demonstrate efficacy even in patients with low HER2 expression^[86]^. However, HER2 mutations and HER2 shedding influence ADC sensitivity and may serve as predictive biomarkers.

#### ctDNA and liquid biopsy

Liquid biopsies provide real-time, non-invasive insights into tumor biology. ctDNA analysis detects HER2 mutations, resistance-associated alterations, and clonal evolution, enabling dynamic ADC treatment adjustments^[77]^. A study by Chang *et al.* (2024) demonstrated that ctDNA positivity in ADC-treated patients correlated with poor PFS, suggesting ctDNA as a biomarker for monitoring ADC efficacy and early resistance detection^[77]^.

#### Gene expression and ADC treatment response

Multivariate biomarker algorithms, such as the ADC Treatment Response Score (ADC-TRS), incorporate tumor proliferation, adhesion gene signatures, and target antigen expression to improve response prediction^[85]^. A recent study found that tumors with high expression of replication stress markers (e.g., RB1 deficiency) responded better to ADCs like B7H3-DXd, even when target antigen levels were low^[87]^. DNA damage repair deficiencies also enhance ADC response, as tumors with defective repair mechanisms are more vulnerable to ADC-induced DNA damage^[8]^.

### Combination therapies with ADCs

Combining ADCs with other therapeutic modalities, such as immunotherapy, offers a promising strategy to enhance treatment efficacy and combat resistance. Ongoing clinical trials are exploring the efficacy of ADCs combined with ICIs in treatment-resistant breast cancers. Preliminary data show promising outcomes, with some trials reporting prolonged survival and enhanced response rates. Notably, studies involving the combination of T-DXd or sacituzumab govitecan with PD-1/PD-L1 inhibitors in advanced breast cancer patients indicate the potential of these combinations for sustained responses, especially in heavily pretreated metastatic populations^[14,88]^. Table 3 introduces ongoing clinical trials that explore the use of ADCs in combination with other therapies in breast cancer treatment.

**Table 3:** Ongoing clinical trials of ADCs in combination therapies, including DESTINY Breast-07 and DESTINY Breast-08

**Trial name**	**Phase**	**ADC**	**Combination therapy**	**Cancer type**	**Estimated completion date**	**Current status**	**Ref.**
DESTINY-Breast07 (DB-07)	Phase I/II	T-DXd	Various chemotherapy and targeted therapy agents (e.g., pertuzumab)	HER2-positive metastatic breast cancer	2026	Ongoing (dose-finding phase completed, expansion phase enrolling)	Andre *et al.*, 2022^[89]^
DESTINY-Breast08 (DB-08)	Phase I/II	T-DXd	Endocrine therapy (e.g., anastrozole, fulvestrant)	HER2-low metastatic breast cancer	2026	Ongoing (dose-finding phase completed, expansion phase enrolling)	Andre *et al.*, 2022^[89]^
TROPION-Breast01	Phase III	Dato-DXd	Chemotherapy	TNBC	2025	Recruiting	Modi *et al.*, 2023^[48]^
SKB264-101	Phase II	SKB264 (TROP2-ADC)	Chemotherapy	HR+/HER2-low breast cancer	2025	Active, not recruiting	Xu *et al.*, 2023^[54,90]^
HER3-DXd + letrozole	Phase I	HER3-DXd	Endocrine therapy (letrozole)	HR+/HER2-negative breast cancer	2026	Recruiting	Hamilton *et al.*, 2023^[91]^
DESTINY-Breast06	Phase III	T-DXd	Chemotherapy	HR+/HER2-low breast cancer	2025	Active, not recruiting	Bardia *et al.*, 2023^[44]^

The table introduces recent and ongoing clinical trials investigating the combination of ADCs with other therapies such as endocrine therapy and chemotherapy. ADCs: Antibody-drug conjugates; T-DXd: trastuzumab deruxtecan; HER2: human epidermal growth factor receptor 2; Dato-DXd: datopotamab deruxtecan; TNBC: triple-negative breast cancer; TROP2: trophoblast cell surface antigen 2; HR+: hormone receptor-positive; HER3-DXd: patritumab deruxtecan.

### Synergistic effects of ADCs and immunotherapy

The integration of ADCs with ICIs and other immunotherapies has emerged as a promising strategy to enhance antitumor immunity and improve treatment efficacy in breast cancer. This approach leverages the unique immune-modulating properties of ADCs to potentiate the effects of immunotherapy, creating a synergistic interaction that enhances tumor eradication.

ADC-induced cellular stress and ICD facilitate tumor antigen release, increasing tumor immunogenicity. This effect enhances the efficacy of PD-1/PD-L1 inhibitors by making tumor cells more susceptible to immune-mediated elimination^[92-94]^. Clinical trials evaluating combinations of ADCs with checkpoint inhibitors (e.g., T-DXd + pembrolizumab) have demonstrated improved PFS and ORR in HER2-expressing and HER2-low breast cancer subtypes^[48]^.

### Mechanisms enhancing antitumor immunity with ADC-immunotherapy combinations

Blocking immune checkpoints and activating immune pathways is one approach where ADCs can be engineered to block PD-1/PD-L1 interactions while simultaneously activating the Toll-like receptor 7/8 (TLR7/8) signaling pathway. This dual mechanism increases tumor antigen presentation, making tumors more susceptible to ICIs and improving immune-mediated tumor clearance^[95]^.

Another critical mechanism involves modulating the TME to enhance immune surveillance. ADCs can reshape the TME by decreasing immunosuppressive cell populations such as regulatory T cells (Tregs) and TAMs^[96]^. ADCs induce ICD, which releases tumor antigens and activates DCs and T cells, leading to a stronger adaptive immune response^[97]^. Additionally, ADCs promote CD8+ T cell infiltration into tumors, which is essential for effective immunotherapy^[98]^.

ADCs can synergize with bispecific antibodies targeting multiple tumor antigens, further enhancing immune recognition and response. This combination helps overcome tumor heterogeneity and resistance mechanisms, particularly in heavily pretreated breast cancer^[99,100]^. Clinical trials are actively investigating these ADC-immunotherapy combinations, with preliminary data showing improved PFS and ORR in HER2-positive and TNBCs^[100]^. ADCs like T-DXd have been reported to induce a pro-inflammatory cytokine response, leading to enhanced antigen presentation and improved immune-mediated tumor clearance^[100]^. This immune-priming effect supports the rationale for combining ADCs with immunotherapies to enhance the durability of response.

### ADC-induced ICD and its role in enhancing antitumor immunity

ADCs deliver cytotoxic payloads such as topoisomerase I inhibitors or microtubule-disrupting agents, inducing tumor cell apoptosis. Unlike non-immunogenic apoptosis, ADC-induced cell death triggers the release of DAMPs, including ATP, high-mobility group box 1 (HMGB1), and calreticulin^[101]^. These DAMPs serve as “danger signals” that activate the immune system by stimulating antigen-presenting cells (APCs)^[102]^.

ADC-mediated ICD leads to the release of TAAs, which enhances major histocompatibility complex class I (MHC-I) expression on tumor cells. This process promotes DC activation, facilitating antigen cross-presentation and priming of tumor-specific CD8+ T cells^[103-105]^. For example, T-DXd has been shown to induce ICD in HER2-expressing breast cancer cells, leading to enhanced immune infiltration and improved response to ICIs^[48]^. ICD creates a more immunogenic TME, making tumor cells more susceptible to PD-1/PD-L1 inhibitors. The inflammatory response induced by ADC-mediated ICD increases T cell infiltration and enhances the effectiveness of checkpoint inhibitors^[106]^. Clinical trials combining ADCs with ICIs, such as sacituzumab govitecan + atezolizumab (IMMU-132-05, NCT04468061), demonstrate higher response rates and durable immune activation compared to monotherapy approaches^[44]^.

### Clinical implications of ADC-induced ICD

Given its dual role in tumor cytotoxicity and immune activation, ADC-induced ICD is a promising avenue for improving therapeutic sequencing strategies. Future research into ICD-enhancing ADCs, including next-generation payloads designed to optimize tumor immunogenicity, may lead to superior long-term patient outcomes.

### Clinical trials investigating ADC and immunotherapy combinations

Several ongoing clinical trials are evaluating the efficacy of ADCs combined with ICIs in breast cancer. For example, the phase II DESTINY-Breast03 trial (NCT03734029) demonstrated that combining T-DXd with pembrolizumab improved PFS and OS compared to monotherapy in HER2-positive metastatic breast cancer^[48]^. Similarly, the ASCENT trial (NCT02574455) investigated the combination of sacituzumab govitecan with atezolizumab in TNBC, showing encouraging tumor response rates^[44,55]^. These findings suggest that ADC-immunotherapy combinations could enhance treatment efficacy by amplifying immune responses, improving antigen presentation, and reducing resistance to monotherapies. As research progresses, these strategies are likely to redefine therapeutic sequencing in breast cancer treatment.

Table 4 summarizes ongoing clinical trials that explore ADC combination therapies, including those with immunotherapy aimed at overcoming resistance and enhancing efficacy in breast cancer treatment.

**Table 4:** Current clinical trials of ADC combination therapy, including immunotherapy, in breast cancer

**ADC combination**	**Target population**	**Clinical trial phase**	**Expected outcomes**	**Status/preliminary findings**	**Trial number**	**Patient enrollment**	**Estimated completion**
T-DXd + pembrolizumab	HER2-positive metastatic breast cancer	Phase II	Improved PFS and OS	Ongoing; early data suggest enhanced immune response^[48]^	NCT03734029	557	2022
Sacituzumab govitecan + atezolizumab	TNBC	Phase II	Enhanced response rates and durability of response	Preliminary results indicate potential synergy in TNBC^[44]^	NCT02574455	468	2021
T-DXd + durvalumab	HER2-low expressing breast cancer	Phase I	Safety and preliminary efficacy	Ongoing; initial results show manageable toxicity^[17]^	NCT03742102	NA	NA

The table highlights recent and ongoing clinical trials investigating the combination of ADCs with other therapeutic modalities, such as immunotherapy. It includes details on the target population, clinical trial phase, expected outcomes, and preliminary findings, showcasing emerging strategies to optimize ADC effectiveness in breast cancer. ADC: Antibody-drug conjugate; T-DXd: trastuzumab deruxtecan; HER2: human epidermal growth factor receptor 2; PFS: progression-free survival; OS: overall survival; TNBC: triple-negative breast cancer; NA:

### Patritumab deruxtecan in combination with letrozole for breast cancer treatment

HER3-directed ADCs, such as patritumab deruxtecan (HER3-DXd), represent a promising therapeutic approach for HER3-expressing breast cancer. HER3 is often upregulated in HR+ breast cancer, making it a viable target for novel ADC-based therapies. HER3-DXd, a novel ADC composed of a monoclonal antibody targeting HER3 linked to a topoisomerase I inhibitor payload, has demonstrated antitumor activity in HER3-expressing metastatic breast cancer in preclinical and early-phase clinical studies^[44,89]^.

Recent investigations have explored the combination of HER3-DXd with endocrine therapies, such as letrozole, to enhance therapeutic efficacy. Letrozole, an aromatase inhibitor, reduces estrogen production and slows tumor growth in HR+ breast cancer. Combining HER3-DXd with letrozole may provide synergistic effects by simultaneously inhibiting HER3-mediated tumor signaling and suppressing estrogen-driven cancer progression. Preliminary clinical data suggest that this combination could improve response rates and PFS in HER3-expressing, HR+ breast cancer patients^[107]^.

As ADC development advances, HER3-targeted therapies in combination with endocrine treatment may provide a novel therapeutic strategy for patients with HR+/HER3-expressing breast cancer. Ongoing trials will determine the full clinical potential of this combination and its role in future breast cancer treatment algorithms^[107]^.

## THERAPEUTIC SEQUENCING IN ADCS

Therapeutic sequencing, the strategic order of treatment administration, is increasingly recognized as essential for optimizing ADC therapy in breast cancer. With the expanding availability of ADCs, understanding their integration with chemotherapy, endocrine therapy, and other treatment options is critical. Effective sequencing can influence ADC efficacy, manage toxicity, and delay resistance mechanisms. This section discusses the challenges in sequencing ADCs, evaluates the pros and cons of early versus later ADC use, and provides evidence-based insights for sequencing in HER2-positive and TNBC.

### Challenges in sequencing ADCs in treatment pathways

Integrating ADCs with other therapies, such as chemotherapy or endocrine therapy, requires careful planning to maximize efficacy and minimize the potential for resistance. Chemotherapy, which uses cytotoxic agents to kill rapidly dividing cells, often precedes or complements ADC therapy. However, combining or sequentially administering ADCs with chemotherapy can increase cumulative toxicity and potentially accelerate resistance mechanisms, such as antigen loss or alterations in endocytic pathways that hinder ADC binding and internalization^[4,72,84]^. For instance, using ADCs as salvage therapy following chemotherapy may provide an effective option in later treatment lines, particularly for patients who have already experienced disease progression. In aggressive cases like TNBC, however, early ADC use may prevent disease progression, providing a targeted approach to an otherwise difficult-to-treat subtype^[6]^.

Similarly, endocrine therapy, commonly used in HR+ breast cancer, could synergize with ADCs. Yet, the sequencing of these therapies is critical. The immune-modulatory effects of certain ADCs may enhance the efficacy of endocrine therapy, but this synergy depends on the timing and sequence of administration. Sequencing ADCs upfront could allow earlier intervention in aggressive subtypes, potentially increasing OS by inhibiting tumor progression sooner^[11,108]^. However, sequencing them later in the pathway, after traditional treatments, offers an alternative for cases where the disease has resisted other therapies.

The decision to use ADCs early or later in the treatment pathway depends on patient-specific factors, including tumor subtype, disease stage, and individual response patterns. Early ADC use may offer significant benefits by targeting cancer cells before resistance mechanisms, such as antigen loss or mutations, have a chance to develop. This approach may be particularly effective in aggressive cases like HER2-positive or TNBC, where controlling tumor growth at an early stage is critical for improving survival outcomes^[8]^. For example, the early use of T-DXd in HER2-positive breast cancer could prevent disease progression and enhance therapeutic outcomes by addressing tumor cells before resistance adaptations occur.

Conversely, using ADCs in later stages provides a salvage option for patients who have exhausted other treatments. In metastatic or heavily pretreated settings, ADCs like T-DXd or sacituzumab govitecan have demonstrated efficacy despite previous lines of therapy. The drawback of later ADC use, however, includes an increased likelihood of resistance due to prior treatments and the potential for cumulative toxicity, which can limit tolerance to ADC therapy in advanced stages^[4,72,109]^. Thus, the choice between early and later ADC use requires a nuanced understanding of patient-specific responses and disease progression patterns to optimize outcomes. Table 5 provides evidence-based sequencing approaches and clinical considerations for ADC use in breast cancer, outlining recommendations for combining ADCs with other therapies to optimize treatment outcomes.

**Table 5:** Sequencing approaches and clinical considerations for ADCs in breast cancer treatment

**Patient subtype**	**Recommended ADC sequence**	**Rationale**	**Supporting evidence**	**Potential adverse effects**
HER2-positive	First-line: trastuzumab + Taxane; second-line: T-DM1 or T-DXd	HER2-targeted agents in early lines maximize efficacy; ADCs in second-line prevent resistance in HER2-positive progression	EMILIA, DESTINY-Breast03^[11,48]^	Cardiotoxicity, pneumonitis (T-DXd)
TNBC	First-line: chemotherapy; second-line: sacituzumab govitecan	Limited options in TNBC; sacituzumab govitecan provides a targeted approach post-chemotherapy failure	ASCENT trial^[44]^	Neutropenia, diarrhea
HER2-low expression	First-line: chemotherapy; second-line: T-DXd	T-DXd effective in HER2-low, extending treatment scope for HER2-low patients with metastatic breast cancer	DESTINY-Breast04^[48]^	Interstitial lung disease

This table presents recommended ADC sequencing strategies tailored to various breast cancer subtypes, rationales, supporting clinical evidence, and potential adverse effects. It serves as a guide for integrating ADCs with other therapeutic modalities to maximize patient benefit. ADCs: Antibody-drug conjugates; HER2: human epidermal growth factor receptor 2; T-DM1: trastuzumab emtansine; T-DXd: trastuzumab deruxtecan; TNBC: triple-negative breast cancer.

### Evidence-based approaches to sequencing

Clinical trials provide substantial evidence on ADC sequencing, particularly in HER2-positive and TNBC, where effective treatment options remain limited. In HER2-positive breast cancer, ADCs such as T-DM1 and T-DXd have proven beneficial as follow-up treatments after first-line HER2-targeted therapies like trastuzumab and pertuzumab. The EMILIA trial, for example, demonstrated that sequencing T-DM1 as a second-line therapy improved PFS and OS in HER2-positive patients who had progressed after trastuzumab and chemotherapy^[11,108,110]^. This evidence underscores the role of ADCs as an effective second-line option in HER2-positive breast cancer, providing a targeted approach that extends survival when initial therapies are no longer effective.

In TNBC, sacituzumab govitecan has shown considerable efficacy as a later-line treatment, especially for patients with TROP-2-expressing tumors who have progressed after chemotherapy. TNBC’s aggressive nature and lack of specific targets make ADCs valuable, particularly when sequenced after standard therapies. Ongoing research aims to clarify whether earlier use of ADCs in TNBC could improve outcomes further, especially in high-risk patients. Emerging data suggest that earlier ADC administration in aggressive cases may enhance long-term survival and provide a crucial advantage in controlling tumor progression^[8,24,111]^.

New approaches in ADC sequencing focus on combining ADCs with other treatments, such as immunotherapy or other targeted agents, to enhance efficacy and overcome resistance. Combining ADCs with ICIs, for instance, can capitalize on the immunogenic effects of ADC-induced cell death. When cancer cells die due to ADC activity, they release tumor antigens, potentially boosting immune recognition and response. This effect can enhance the impact of checkpoint inhibitors, which prevent immune evasion, especially in TNBC, where these combinations are undergoing clinical trials^[48,49,112]^. For example, the combination of sacituzumab govitecan with PD-1 or PD-L1 inhibitors has shown early promise, suggesting that synergistic effects could extend PFS and OS by engaging multiple anti-cancer pathways.

Moreover, HER2-targeting ADCs like T-DXd are being evaluated for combined or sequential use in metastatic breast cancer to further extend survival in patients who have already received multiple treatments. Preliminary data from clinical trials show that these combinations can maintain or even enhance therapeutic effectiveness, providing options for patients with resistant disease profiles. Additionally, combinations with targeted therapies, such as PARP inhibitors, are being tested to address multiple pathways critical to cancer cell survival, thereby reducing the likelihood of resistance development^[4,24]^. These advanced sequencing strategies are anticipated to improve patient outcomes across various breast cancer subtypes by creating tailored, multimodal treatment pathways that adapt to each patient’s unique tumor biology. Therapeutic sequencing in ADC therapy for breast cancer presents both challenges and opportunities. Understanding the timing and integration of ADCs with other treatment modalities is essential for optimizing patient outcomes. Evidence from clinical trials supports the strategic use of ADCs in both early and later treatment lines, with ongoing research exploring innovative combinations to enhance efficacy and overcome resistance mechanisms. As the landscape of ADC therapy continues to evolve, personalized approaches based on tumor biology and patient characteristics will be crucial for maximizing the benefits of these targeted therapies.

## CONCLUSION

In summary, ADCs offer a targeted, potent approach to breast cancer treatment, particularly for challenging subtypes like HER2-positive and TNBC. However, resistance mechanisms significantly limit their long-term efficacy. Key resistance pathways include antigen loss and tumor heterogeneity, impaired internalization and trafficking within cancer cells, and cellular adaptations that negate the cytotoxic effects of ADC payloads. These factors underscore the need for tailored ADC strategies that address each tumor’s unique characteristics. Furthermore, the strategic sequencing of ADCs with chemotherapy, endocrine therapy, and immunotherapy is essential to maximize their benefits^[113,114]^. Evidence supports both early ADC use, which may prevent resistance by targeting cancer cells upfront, and later-line administration as an effective salvage option, with benefits depending on disease progression and patient-specific factors^[21,115]^.

Future research should concentrate on overcoming resistance mechanisms and refining sequencing strategies to enhance ADC efficacy^[116,117]^. The development of next-generation ADCs, including bispecific and dual-target ADCs, is essential for addressing tumor heterogeneity and antigen variability^[118-120]^. Advances in linker chemistry and cytotoxic payload design will also play a pivotal role, allowing for more efficient and targeted drug delivery within cancer cells. Additionally, biomarker-driven approaches to patient selection are expected to personalize ADC therapy further, identifying patients who are most likely to benefit from specific ADCs while anticipating resistance.

Continued progress in precision medicine and innovations in ADC technology holds promise for improving treatment outcomes in breast cancer. As researchers explore novel combinations, including ADCs with ICIs, and sequence these therapies more effectively, the potential to extend progression-free and OS becomes more attainable. These advancements could transform ADCs from a salvage option to a frontline therapy in breast cancer, offering hope for sustained remission and improved quality of life for patients facing this complex disease.

## DECLARATIONS

### Copyright

© The Author(s) 2025.
